# Potential biomarkers for MCL1 inhibitor sensitivity

**DOI:** 10.46439/signaling.2.046

**Published:** 2024

**Authors:** Lei Duan, Carl G. Maki

**Affiliations:** 1 Department of Anatomy and Cell Biology, Rush University Medical Center, Chicago, IL 60612, USA

**Keywords:** MCL1 inhibitor, Triple negative breast cancer, Predictive marker, AXL, ETS1, IL6, EFEMP1

## Abstract

MCL1 is an anti-apoptotic member of the BCL2 protein family, and its overexpression is associated with poor prognosis across various cancers. Small molecule inhibitors targeting MCL1 are currently in clinical trials for TNBC and other malignancies. However, one major challenge in the clinical application of MCL1 inhibitors is the inherent or acquired resistance to these drugs. Additionally, there is a lack of predictive biomarkers to identify which tumors will respond to MCL1 inhibition. We identified a four-gene functional signature that promotes MCL1 inhibitor resistance in TNBC cells. This gene signature (GS) can distinguish resistant from sensitive TNBC cell lines. Factors encoded by these four genes promote MCL1 inhibitor resistance at least in part through regulation of the ERK signaling pathway. This mechanism involves the upregulation of BCL2 and the downregulation of BIM, which contribute to the inhibitor resistance. Thus, we have discovered a functional GS that drives MCL1 inhibitor resistance. Currently, the MCL1 inhibitor GS-9716 is in clinical trials for TNBC therapy. If validated in clinical samples, this GS could potentially serve as a predictive biomarker for therapy response and help guide the selection of combination therapies to enhance the effectiveness of MCL1 inhibitors.

## Challenges for Clinical Development of MCL1 Inhibitors

Myeloid cell leukemia-1 (MCL1) is an anti-apoptotic member of the B-cell lymphoma 2 (BCL-2) family and plays a key role in cancer cell survival and resistance to therapy [1,2]. MCL1 is often overexpressed in cancers such as leukemia, lymphoma, and solid tumors, allowing cancer cells to evade apoptosis and resist conventional treatments. This makes MCL1 an attractive therapeutic target for cancer therapy.

MCL1 promotes survival by binding the BH3 domains of pro-apoptosis BCL2 family members and inhibiting their activity. BAX and BAK oligomers form pores at the mitochondria outer membrane that causes release of cytochrome C, activation of caspases, and apoptotic death. MCL1 binds tightly to the BH3 domains of BAX and BAK, thus sequestering BAX and BAK proteins and inhibiting their oligomerization. Activator BH3 only proteins such as Noxa and BIM can bind directly to BAX and BAK to stimulate their apoptotic function at the mitochondria. MCL1 binds and sequesters these BH3-only proteins to inhibit their activity. Notably, binding between MCL1 and BH3-only proteins can also have the opposite effect. That is, BH3-only protein binding to MCL1 can also sequester MCL1 and prevent MCL1 binding to BAX and BAK. In cancers where MCL1 is overexpressed, the excess levels of MCL1 protein can overwhelm the BH3-only proteins while still maintaining inhibition of BAX and BAK and, in this way, promote survival. MCL1 inhibitors are designed to mimic BH3 domains and occupy the BH3-domain binding groove in MCL1, in this way blocking MCL1 interaction with the BH3 domains of other proteins and inducing cancer cell death [1,2]. A number of MCL1 selective inhibitors have been developed and are in various stages of development [3]. These inhibitors, including AMG 176, AMG 397, AZD5991, S63845, S64315, ABBV-467, PRT1419, and GS-9716, have shown promising preclinical activity, particularly in cancers resistant to other treatments. Many of these inhibitors are undergoing clinical trials, particularly for hematologic malignancies and solid tumors. For example, AMG 176 and AMG 397 have been evaluated in phase I trials for relapsed or refractory multiple myeloma and acute myeloid leukemia. Similarly, S64315 and AZD5991 have completed studies assessing safety and efficacy in patients with hematologic cancers. Most recently, GS-9716 is being evaluated in phase I trials for advanced solid tumors, both as a monotherapy and in combination with other anticancer therapies.

Despite their promise, the development of MCL1 inhibitors faces significant challenges. Achieving selective inhibition of cancer cells without affecting healthy cells and tissues remains difficult, and many MCL1 inhibitors have short half-lives. Additionally, cancer cells may exhibit intrinsic and acquired resistance to MCL1 inhibition that could reduce the clinical efficacy of MCL1 inhibitors. Off-target toxicity, particularly cardiac toxicity, is another concern. For example, the FDA placed a clinical hold on AMG 397 due to cardiac toxicity concerns. AZD5991 was also linked to high troponin levels and low response rates in phase I trials, resulting in its study closure [3]. Overcoming these challenges is essential for translating the preclinical success of MCL1 inhibitors into effective clinical cancer therapies.

## Discovery of Potential Markers and Functional Mechanisms of Resistance

How can one overcome the dose-limiting toxicity associated with MCL1 inhibitors, and how can one overcome the intrinsic resistance observed in some cancer cells? The most direct approach for reducing toxicity is to use a lower inhibitor dose. However, this approach is problematic as lower doses, while reducing toxicity, may also have lower anti-tumor activity. A promising alternative is to identify predictive biomarkers that can identify tumors that are especially sensitive to MCL1 inhibitors, even at low doses. Most biomarkers in current clinical use are driver mutations in oncogenes, which are directly targeted with specific drugs. Due to oncogene addiction, tumors harboring these mutations often respond significantly better to targeted treatments than healthy cells, resulting in clinical efficacy with manageable toxicity for patients. An example is the KrasG12C mutation in NSCLC, which renders tumors harboring this mutation (but not normal cells and tissues) sensitive to the recently approved KrasG12C inhibitor sotorasib. However, for non-mutated targets such as MCL1, the lack of molecular markers that indicate sensitivity poses a substantial challenge. Some cancers have elevated levels of MCL1 protein and are resistant to therapy. However, the level of MCL1 expression itself does not correlate with MCL1 inhibitor response in cancer cells. Thus, cells with relatively high levels of MCL1 expression can be resistant to MCL1 inhibitor, and cells relatively low levels of MCL1 expression can be sensitive, indicating MCL1 expression is an imperfect predictor of MCL1 inhibitor sensitivity [4]. A question then arises – how can one identify potential markers of MCL1 inhibitor response that then could be used to stratify patients that would likely most benefit from treatment?

We approached this problem in two ways. First, we carried out cell-based studies to identify triple negative breast cancer cell lines more or less sensitive to MCL1 inhibitor treatment. We then used informatics approaches that included the CCLE, GDSC, and DepMap databases. Through these analyses we identified 4 genes that are expressed at elevated levels in TNBC cells resistant to the MCL1 inhibitor S63845, and expressed at low levels in TNBC cells that were especially sensitive to S63845. Our goal then was to determine whether this 4 gene set could be used as a biomarker to identify TNBC cells that would be most sensitive. To achieve this, we developed a gene signature (GS) score for cells based on the relative expression levels of these four genes and performed a ROC curve analysis to evaluate its potential as a predictive biomarker. TNBC cell lines were classified as either sensitive or resistant based on their response to the MCL1 inhibitor AZD5991, and as MCL1-dependent or -independent based on their Chronos scores (DepMap). We then analyzed the correlation between the GS scores and resistance/independence using the ROC curve method (IBM SPSS software), with resistance and independence defined as event 1. Our results demonstrated that the GS score is a highly robust predictor of MCL1 inhibitor sensitivity in TNBC cells, with a ROC area score of 0.948 (where a score of 1 indicates perfect correlation) [4].

**Figure 1.**
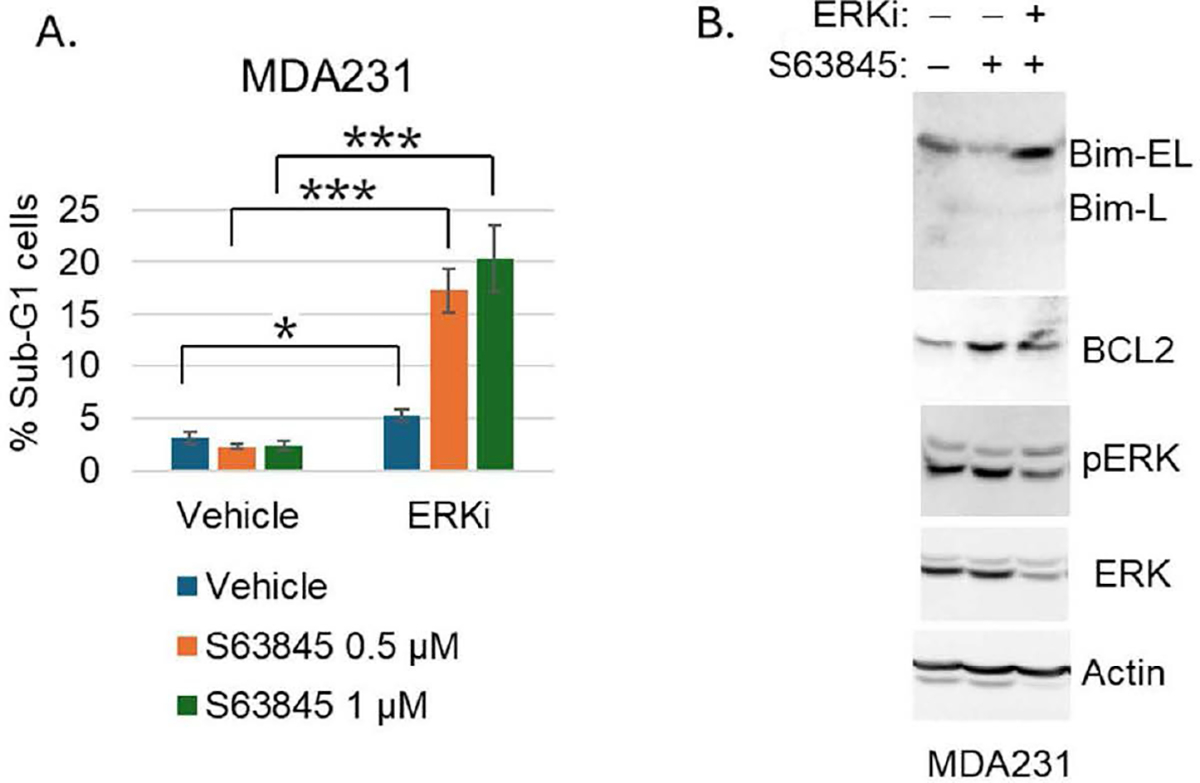
ERK Inhibition Sensitizes MCL1-Resistant MDA231 Cells to S63845 by Modulating BIM and BCL2 Levels. We hypothesize that the gene signature (GS) promotes resistance, in part, through ERK-mediated upregulation of BCL2 and downregulation of BIM. To test this, we treated MCL1 inhibitor-resistant MDA231 cells with S63845, with or without the ERK-specific inhibitor ulixertinib, followed by FACS analysis of the cell cycle (Sub-G1 for apoptosis) and immunoblot analysis of BIM and BCL2 protein expression. **A.** MDA231 cells were treated with vehicle, S63845 (0.5 and 1 μM), or S63845 in combination with ulixertinib (ERKi, 2 μM) for 3 days. Cells were analyzed by FACS for cell cycle distribution. The percentage of Sub-G1 (apoptotic) cells from triplicate samples is shown, with standard deviation (SD) and statistical significance indicated (* p<0.05, ***p<0.0005). **B.** Cells were treated with vehicle, S63845 (1 μM), or S63845 in combination with ulixertinib (2 μM) for 48 hours. Cell lysates were subjected to immunoblot analysis for the indicated proteins.

What are the 4 genes and what are the proteins they encode? The 4 genes are *AXL, EFEMP1, IL6,* and *ETS1.* AXL is a receptor tyrosine kinase that activates the RAS-MEK-ERK pathway downstream, and IL-6 and EFEMP1 are ligands that bind their receptors and also activate RAS-MEK-ERK [5–9]. The fourth factor (ETS1) is a transcription factor that we found can stimulate expression of AXL, IL-6, and EFEMP1 to activate ERK, and that is also phosphorylated and activated by ERK [4]. Thus these 4 factors function in pathways that activate ERK downstream. ERK is particularly interesting because previous studies showed activated ERK can promote expression of BCL2 and BCLxL while inhibiting expression of pro-apoptotic BIM, and high expression of BCL2 and BCLxL promotes MCL1 inhibitor resistance [3,10,11]. Moreover, several studies reported ERK inhibitors when combined with MCL1 inhibitors can enhance cancer cell killing [12–14]. We speculated inhibition of these 4 factors, or direct inhibition of ERK, would cause synergistic killing in TNBC cells when co-treated with MCL1 inhibitor. Indeed, our studies showed inhibition or knockdown of AXL, IL-6, EFEMP1, and ETS1 could stimulate MCL1-inhibitor induced killing in otherwise resistant TNBC cells [4]. Currently, we carried out a further analysis to ask if direct inhibition of ERK would have the same effect, as shown in the Figure, direct inhibition of ERK by ulixertinib caused synergistic killing when combined with MCL1 inhibitor of the otherwise resistant MDA231 cells (Figure 1A). Notably, in MCL1 inhibitor-resistant MDA231 cells, MCL1 inhibition resulted in downregulation of BIM while increasing BCL2 protein levels, without altering ERK phosphorylation (Figure 1B). This suggests that the constitutively active ERK signaling plays a key role in regulating the balance between BIM and BCL2, thus promoting cell survival in response to MCL1 inhibition. Inhibition of ERK reverses this effect, restoring BIM expression and reducing BCL2 levels (Figure 1B), ultimately leading to cell death.

Our results suggest that the expression levels of the four genes we identified can serve as predictive markers for MCL1 inhibitor sensitivity in TNBC cell lines. Additionally, the factors encoded by these genes promote resistance to MCL1 inhibitors and could be targeted to overcome this resistance. As previously mentioned, a major challenge in the clinical use of MCL1 inhibitors is dose-limiting toxicity, highlighting the urgent need for biomarkers to stratify patients and identify those most likely to benefit from treatment.

In our analysis, we identified a four-gene signature that appears to meet this need. We also discovered that the factors encoded by these genes regulate ERK signaling and the expression of BIM and BCL2. Inhibition of these factors, or direct inhibition of ERK, stimulates BIM expression and sensitizes cells to MCL1 inhibitors. Based on these findings, we propose that this gene signature functions as a regulatory marker of ERK signaling, influencing MCL1 inhibitor resistance (see proposed model).

We believe that this gene signature could have a significant impact on ongoing clinical trials and the potential use of MCL1 inhibitors in clinical settings. However, further validation in patient samples is necessary, as no clinical trial data is currently available for this purpose. Additionally, whether this gene signature can be applied to other cancer types remains to be tested in future studies.

## Figures and Tables

**Proposed Model.**
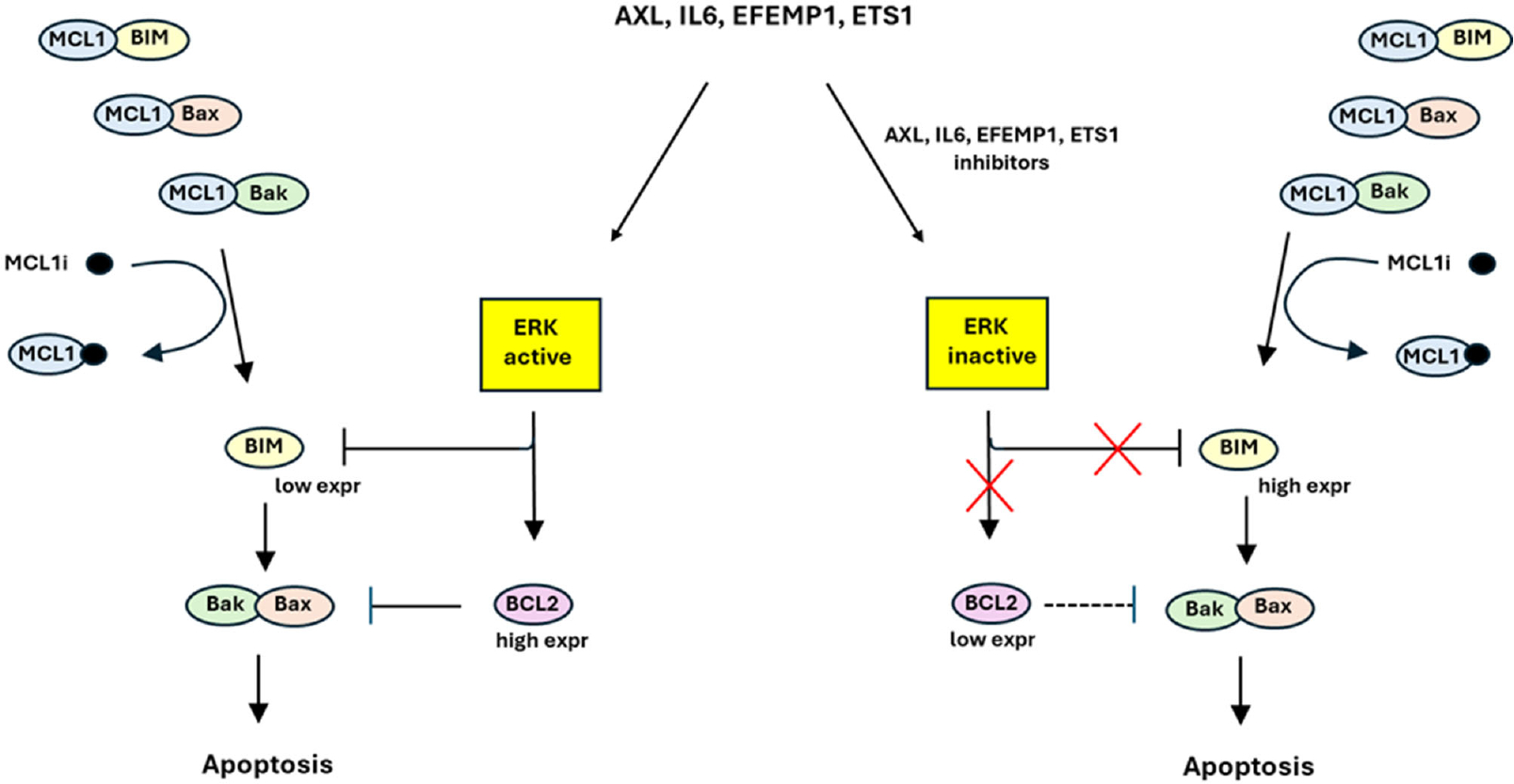
AXL, IL6, EFEMP1, and ETS1 constitute a 4-member gene signature that predicts MCL1 inhibitor sensitivity and promotes MCL1 inhibitor resistance. **(Left)** MCL1 inhibitor (MCLi) sequesters MCL1, releasing BIM, Bax, and Bak to promote apoptosis. However, these effects are countered by ERK activity downstream of AXL, IL6, EFEMP1, and ETS1 that inhibits BIM expression and increases BCL2. **(Right)** Inhibitors of AXL, IL6, EFEMP1, and ETS1 (or direct ERK inhibitors) reduce ERK activity, thus increasing BIM expression and reducing BCL2 and sensitizing cells to MCL1 inhibitor. Low expression of AXL, IL6, EFEMP1, and ETS1 identifies TNBC cells that are sensitive MCL1 inhibitor; high expression of these 4 factors identifies cells that are resistant.
